# Oral Probiotic Control Skin Inflammation by Acting on Both Effector and Regulatory T Cells

**DOI:** 10.1371/journal.pone.0004903

**Published:** 2009-03-20

**Authors:** Feriel Hacini-Rachinel, Hanane Gheit, Jean-Benoit Le Luduec, Fariel Dif, Stéphane Nancey, Dominique Kaiserlian

**Affiliations:** 1 Université de Lyon, Lyon, France; 2 INSERM, U851, Lyon, France; 3 Université de Lyon1, IFR128, Lyon, France; 4 Danone Research, Palaiseau, France; 5 Hospices Civils de Lyon, Service de Gastroentérologie, Centre hospitalier Lyon Sud, Lyon, France; New York University School of Medicine, United States of America

## Abstract

Probiotics are believed to alleviate allergic and inflammatory skin disorders, but their impact on pathogenic effector T cells remains poorly documented. Here we show that oral treatment with the probiotic bacteria *L. casei* (DN-114 001) alone alleviates antigen-specific skin inflammation mediated by either protein-specific CD4^+^ T cells or hapten-specific CD8^+^ T cells. In the model of CD8^+^ T cell-mediated skin inflammation, which reproduces allergic contact dermatitis in human, inhibition of skin inflammation by *L. casei* is not due to impaired priming of hapten-specific IFNγ-producing cytolytic CD8^+^ effector T cells. Alternatively, *L. casei* treatment reduces the recruitment of CD8^+^ effector T cells into the skin during the elicitation (*i.e.* symptomatic) phase of CHS. Inhibition of skin inflammation by *L. casei* requires MHC class II-restricted CD4^+^ T cells but not CD1d-restricted NK-T cells. *L casei* treatment enhanced the frequency of FoxP3^+^ Treg in the skin and increased the production of IL-10 by CD4^+^CD25^+^ regulatory T cells in skin draining lymph nodes of hapten-sensitized mice. These data demonstrate that orally administered *L. casei* (DN-114 001) efficiently alleviate T cell-mediated skin inflammation without causing immune suppression, via mechanisms that include control of CD8^+^ effector T cells and involve regulatory CD4^+^ T cells. *L. casei* (DN-114 001) may thus represent a probiotic of potential interest for immunomodulation of T cell-mediated allergic skin diseases in human.

## Introduction

Probiotics are defined by FAO and WHO as live microorganisms which when administered in adequate amounts, confer a health benefit to the host [1]. Orally administered probiotics exhibit widespread effects on gut homeostasis and immunomodulation of both mucosal and systemic immunity. Probiotics may counterweight aggressive enteric commensals in the gut and reinforce the barrier function of the epithelium but can also contribute in the regulation of innate and adaptive immune responses of the host under healthy or pathogenic conditions (reviewed in [2]). Lactic acid bacteria including bifidobacteria and lactobacilli used as probiotics are commensal bacteria of the gut microbiota that could be used for prevention or treatment of chronic allergic and inflammatory diseases, such as inflammatory bowel disease (IBD) [3] and atopic dermatitis [4]. Recently, studies in patients with IBD [5] and in animal models of colitis [6], [7] emphasized that probiotics modulate the outcome and severity of intestinal inflammation. Oral administration of a probiotic cocktail (VSL#3) composed of several lactic acid bacteria reduced the severity of chronic T-cell mediated TNBS induced-colitis in mice by acting via a subset of TGFß-bearing cells in the gut *lamina propria* [6]. In a recently developed mouse model of colitis mediated by cytotoxic CD8 T cells [8], we observed that *L. casei* DN-114 001 exerts a protective effect on the severity of intestinal lesions and enhances the function of mucosal CD4^+^ FoxP3^+^ Treg in the colon *lamina propria* (Hacini-Rachinel *et al*, submitted).

Besides immunomodulation on mucosal inflammation, probiotic may also regulate systemic immune responses that contribute to allergic and inflammatory skin diseases. Studies in human have documented that atopy including atopic dermatitis is less frequently observed in children from mothers who have been under a probiotic diet during pregnancy and the post natal period [9]. The outcome of probiotic treatment on systemic immune responses involved in chronic inflammatory and allergic diseases is still poorly documented. Contact dermatitis is one of the most common skin allergies in western countries [10]. We previously reported that oral administration of *L. casei* (strain DN-114 001)-fermented milk reduces T cell-mediated delayed-type contact hypersensitivity (CHS) to the hapten 2-4-dinitrofluorobenzene (DNFB) in normal mice [11], which reproduces the pathophysiology of allergic contact dermatitis in human. Moreover, a clinical study in human with allergic contact dermatitis to Nickel showed that oral treatment with *L. casei*-fermented milk reduces the severity of prurit and itching (*Danone patent EP 1 838 158 B1*). However, the impact and mode of action of probiotics, including *L. casei*, on immune responses involved in the pathophysiology of allergic diseases remains elusive.

Development of allergic contact dermatitis to Nickel [12], drugs (*i.e.* antibiotics) and contact sensitizing chemicals (*i.e.* food conservative, perfume, dyes) [13] requires two distinct immunological phases. The asymptomatic sensitization phase (*i.e.* the afferent phase of CHS), which is induced by topical skin exposure to the hapten, results in the priming of hapten-specific T cells. The symptomatic phase (*i.e.* the efferent phase of CHS) is elicited upon a novel exposure to the same hapten in sensitized individuals and leads within 24/48 hours to the recruitment of the inflammatory skin infiltrate and induction of skin lesion. We and others have extensively characterized the pathophysiology of the CHS response to DNFB in mice and showed that skin inflammation is mediated by hapten-specific IFNγ-producing Tc1-type CD8^+^ T cells primed within 5 days in skin draining lymph nodes [13], [14], [15], [16]. These CD8 effectors initiate the inflammatory process by performing Fas/Fas ligand and perforine-mediated apoptosis of keratinocytes [17], [18]. *In vivo* priming of CD8 effectors and development of the CHS response are independent of CD4 T cell help and the intensity and resolution of skin inflammation is under control of regulatory CD4 T cells (Treg) [15], [19]–[21] including CD4^+^CD25^+^FoxP3^+^ Treg [22]. Studies in human with Nickel allergy have confirmed that skin inflammation is mediated by Tc1-type CD8 effectors and regulated by CD4^+^CD25^+^ Treg [23].

In the present study, using experimental models of DTH responses mediated by CD4^+^ T cells specific to a protein Ag, ovalbumin) and by CD8^+^ T cells specific to the hapten DNFB, we found that oral administration of live *L. casei* strain DN-114 001 down-regulate skin inflammation mediated by CD4^+^ or CD8^+^ T cells. We demonstrated in the CHS model that *L casei* does not impair the priming or differentiation of hapten-specific CD8 T cells in lymphoid organs, but reduces the frequency of CD8 T cells recruited into the skin upon elicitation of CHS by hapten challenge. We show that this effect requires CD4^+^ T cells and is accompanied by an effect of *L. casei* on the frequency and function of Treg.

## Materials and Methods

### Mice

Female C57Bl/6 (2–4 month old) were purchased from Charles River laboratories (L'Arbresle, France). MHC-class II (Aβ°/°)-deficient mice were kindly provided by D. Mathis and C. Benoist, CD1d°/° by L. VanKaer, CD3ε°/° by M. Malissen and bred as homozygotes (C57Bl/6 back-ground, 9^th^ generation) in our animal facilities. All mice were bred at the institute's animal facilities (Plateau de Biologie Expérimentale de la Souris, Ecole Normale Superieure de Lyon) under specific pathogen-free conditions. All experiments were previously approved by the Animal Care and Use Committee according to governmental guidelines.

### Bacteria

*L. casei* DN-114 001 was grown for 16 h at 37°C under aerobic conditions in neutral Man-Rogosa-Sharpe liquid medium (MRS broth, Difco, France). Bacteria were harvested in the stationary phase of growth and stored at 4°C. Viability of *L. casei* DN-114 001 was >95% for up to 1 week at 4°C. Just before use, bacteria were washed twice in 0.9% NaCl and adjusted at 10^8^ cfu/ml.

### Probiotic treatment

Treatment was started at day –14 before epicutaneous sensitization with DNFB and continued until the end of the experiments (i.e. day 12 after sensitization). Mice (5–7 per group) were fed daily by an intragastric stainless steel feeding tube with 200 µl of either live *L. casei* DN-114 001 (10^8^ cfu/ml), or sterile 0.9% NaCl (control).

### Contact hypersensitivity (CHS) to DNFB

Contact sensitivity to DNFB was induced as described [21]. Briefly, mice were sensitized on day 0 by epicutaneous application onto the shaved abdomen of 25 µl of 0.5% DNFB (Sigma Aldrich, St. Quentin Fallavier, France) diluted in acetone/olive oil (4∶1, vol/vol) (i.e. vehicle). On day 5, mice were ear challenged with 0.15% DNFB applied onto the right ear; the left ear received the vehicle alone. Ear thickness was measured with a micrometer (J15 Blet, Lyon, France) before and each day after challenge. Ear swelling was calculated as the difference of swelling between the right and the left ear (ear swelling = ear thickness after challenge – ear thickness before challenge). Results are expressed in µm. Ear swelling after DNFB challenge in unsensitized mice was routinely below 20 µm.

### Delayed Type Hypersensitivity (DTH) to ovalbumin

Mice were sensitized subcutaneously with 50 µg of OVA (grade VII, Sigma, France) in 25 µl of saline emulsified with an equal volume of CFA and injected in both sides of the base of the tail. Seven days later the DTH response was elicited by sc challenge with 200 µg of aggregated OVA injected *s.c.* into the left hind footpad, while the right hind footpad was injected with saline. Aggregated OVA was prepared by heating a 2% solution of OVA at 70°C for 1 h. After cooling, the precipitate was washed and re-suspended in the original volume of saline. Footpad thickness was measured at 24 and 48 h after challenge using calipers. Footpad swelling was determined by subtracting values given by saline-injected footpads from those of Ag-injected footpads.

### Isolation of CD8^+^ T cells and CD4^+^ CD25^+^ T cells

After B cells depletion using columns coated by goat anti-mouse and anti-rat IgG BioMag® beads (Qiagen, Germany), CD8^+^ T cells from splenic, axillary and inguinal lymph nodes of day 5 hapten-sensitized mice were purified by positive selection using anti-CD8 mAb-coated microbeads and magnetic columns (Miltenyi Biotec, France). CD4^+^CD25^+^ T cells were purified by positive selection using anti-CD25 mAb-coated microbeads. The percentage of CD8^+^ T cells and CD4^+^CD25^+^ T cells was routinely >90% and 70% respectively as determined by FACS analysis.

### Direct *in vivo* cytotoxicity assay

*In vivo* analysis of hapten-specific cytotoxic T cells (CTL) was assessed on day 5 after immunization, as described [8]. Mice were injected *i.v.* with a mixture of target cells including, 10 10^6^ DNBS-pulsed and 10 10^6^ unpulsed spleen cells, previously labeled with 0.5 µmol/L and 5 µmol/L of carboxy-fluorescein succinimidyl ester (CFSE), respectively. 24 hours later, cell suspensions from pLNs and spleens were prepared and 10,000 CFSE positive cells were acquired by using a FACSCalibur (BD Biosciences, San José, CA). *In vivo* cytotoxicity was assessed by calculating the ratio of unpulsed/pulsed targets in immunized versus naive mice.

### Extraction of skin cells

Total ear cell suspensions were prepared as previously described [24]. Ears were split into dorsal and ventral halves, cut in small pieces and incubated for 90 min at 37°C with 400 U/ml of collagenase IA (Sigma) and 400 U/ml of DNAse I (Roche Diagnostics, Germany) in RPMI 1640 supplemented with 10% FCS, 25 mM Hepes. Cell suspensions were filtered through nylon mesh (100 µm) washed and resuspended in PBS containing 2% FCS, 0.5 mM EDTA and 0.01% NaN_3_ and stained for FACS analysis.

### Flow cytometry analysis

Cells were first incubated with 2.4G2 hybridoma supernatant to block Fcγ receptors and then stained with various combinations of the following anti-mouse antibodies from BD Pharmingen : allophycocyanin (APC)-conjugated rat anti- CD45 (IgG2b,k; clone 30-F11), peridinin chlorophyll protein-cyanin 5.5 (PerCP-Cy5.5)-conjugated rat anti-CD8a (IgG2a,k; clone53-6.7), PerCP-conjugated rat anti-CD4 (IgG2a,k; clone RM4-5); fluorescein isothiocyanate (FITC)-conjugated rat anti-CD25 (IgM; clone 7D4). Appropriate isotype-matched mAbs were used as control. For intracellular Granzyme B staining cells were fixed and permeabilized with Cyto-fix-Cytoperm (BD, France) and stained with the mouse anti-human granzyme B-phycoerythrin (PE) (Caltag). Rat anti-mouse FoxP3 (IgG2a,k; clone FJK-16s) staining was performed according to the manufacturer' instructions (eBiosciences). Fluorescence was measured with a FacsCalibur equipped with CellQuest software (BD, Canada).

### CHS response in the T cell transfer model in CD3ε KO mice

CD8^+^ T cells (5.10^6^) alone or together with CD4^+^CD25^+^ T cells (0,5.10^6^) from C57Bl/6 14 days were isolated after 14 days oral treatment with *L. Casei* and transfered *i.v* into CD3ε°/° mice one day before skin sensitization with 0,5% DNFB. Mice were ear challenged 5 days later with 0,14% DNFB and the CHS response was determined as described above.

### IL-10 production

CD4^+^ CD25^+^ T cells (2. 10^5^) from day 5 DNFB-sensitized mice were cultured for 72 h with soluble 1 µg/ml of anti-mouse CD3 (BD Pharmingen, France) with 10 U/ml of murine IL-2 (Roche Diagnostics, Germany). Supernatants were titrated for IL-10 using an ELISA kit (R&D, USA).

### Statistical analysis

Statistical analyses were perfomed using th Student's *t*-test and the non-parametric Mann-Whitney two-sample test, using a two-sided significance level of 5%. P values<0.05 were considered to be statically significant (*: 0.01 to 0.05, **: 0.001 to 0.01, ***: <0.001).

## Results

### Oral *L. casei* reduces systemic CD4^+^ and CD8^+^ T cell-mediated DTH responses

**Figure 1:**
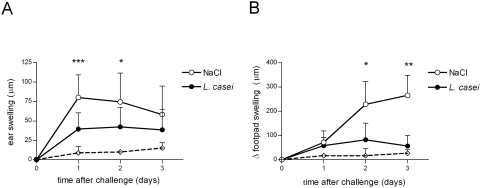
Immunomodulatory effect of *L. casei* on DTH responses. C57Bl/6 mice were treated daily with either live *L. casei* (black circle) or physiologic water (white circle) from day –14 before sensitization until the end of the experiment. (A) CHS to DNFB was induced by skin painting on day 0 with 0.5% DNFB and ear challenge on day 5 with 0.15% DNFB (right ear) or the vehicle alone (left ear). The CHS response was determined by ear swelling at various time points after challenge, as described in Material and Methods. The dotted line represents ear swelling after ear painting with 0.15% DNFB in unsensitized mice. (B) DTH to OVA was induced by subcutaneous injection on day 0 with 50 µg OVA mixed with CFA and footpad challenge 7 days later with either 250 µg aggregated ovalbumin (right footpad) or physiologic water (left footpad). The DTH response was determined by footpad swelling at various time points after challenge, as described in Material and methods. The dotted line represents foodpad swelling after footpad challenge with OVA in mice immunized with CFA alone. Results are expressed as mean±SD of swelling for 7–10 mice per group. Data are representative of three to ten experiments. *p* values were performed using the Mann-Whitney two-sample and Student's t-tests.

We tested whether oral treatment with *L. casei* could alleviate systemic DTH responses mediated by Ag-specific T cells in C57Bl/6 mice. Mice treated daily by gavage from day –14 with 2.10^7^ cfu of live *L. casei* DN 114001, exhibited a reduced CHS to DNFB (**Fig 1A**). *L. casei*-treatment also decreased the skin DTH response to OVA as demonstrated by the decreased footpad swelling reaction (**Fig 1B**). Thus, oral administration of live *L. casei* into normal mice reduces the severity of T cell-mediated DTH responses mediated by Ag-specific CD4^+^ or CD8^+^ T cells.

### *L. casei* does not impair *in vivo* priming of hapten-specific CD8^+^ CTL effectors

**Figure 2:**
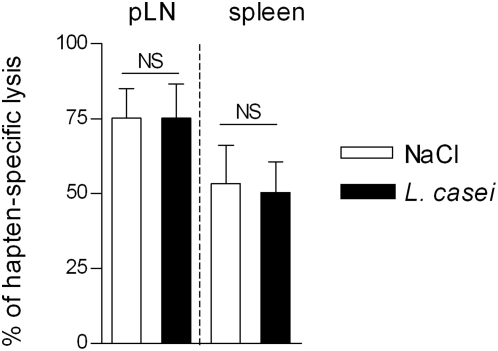
Effect of *L. casei* on hapten-specific CD8^+^ CTL *in vivo*. Hapten-specific *in vivo* CTL assay was carried out in mice treated daily from day -14 with either NaCl (white bars) or *L. casei* (black bars). Mice were sensitized on day 0 with 0.5% DNFB and 5 days later were injected intravenously with a 1∶1 mixture of DNBS-pulsed and unpulsed spleen cells as target cells, stained with 0.5 µmol/L and 5 µmol/L of CFSE, respectively. Twenty-four hours later, the percentage of stained cells in pooled pLN and spleen was analyzed by FACS. In vivo cytotoxicity was calculated by determining the ratio of control targets/pulsed targets. Results are expressed as mean±SD of the percentage of hapten-specific cytotoxicity.

We previously documented that hapten-specific IFNγ-producing cytolytic CD8^+^ T cells primed in skin draining LN during the afferent phase of CHS represent the pathogenic effectors of the CHS response to DNFB [17]. We thus investigated whether *L. casei* affected the *in vivo* priming of Ag-specific CD8^+^ T CTL induced by skin sensitization, using a direct *in vivo* cytotoxic assay. Mice were injected on day 5 after sensitization with a mixture of hapten-pulsed and unpulsed target cells labeled with a low and high concentration of the fluorescent marker CFSE, respectively and their fate in lymphoid organs was analyzed the next day by flow cytometry. As shown in **Fig 2**, control NaCl-treated and *L. casei*-treated mice exhibited on day 5 after sensitization a similar DNFB-specific CTL activity in skin draining LN (NaCl treated: 75.23%±9.86; *L. casei* treated group: 75.22%±11.28) and spleen (NaCl treated: 53.38%±12.81; *L. casei* treated group: 50.26%±10.37). Thus, *L. casei* oral administration did not impair the *in vivo* priming and differentiation of hapten-specific specific CD8^+^ T cells into functional cytolytic CHS effectors in skin draining LN during the afferent phase of CHS.

### *L. casei* reduces recruitment of CD8^+^ CHS effectors into the skin

**Figure 3:**
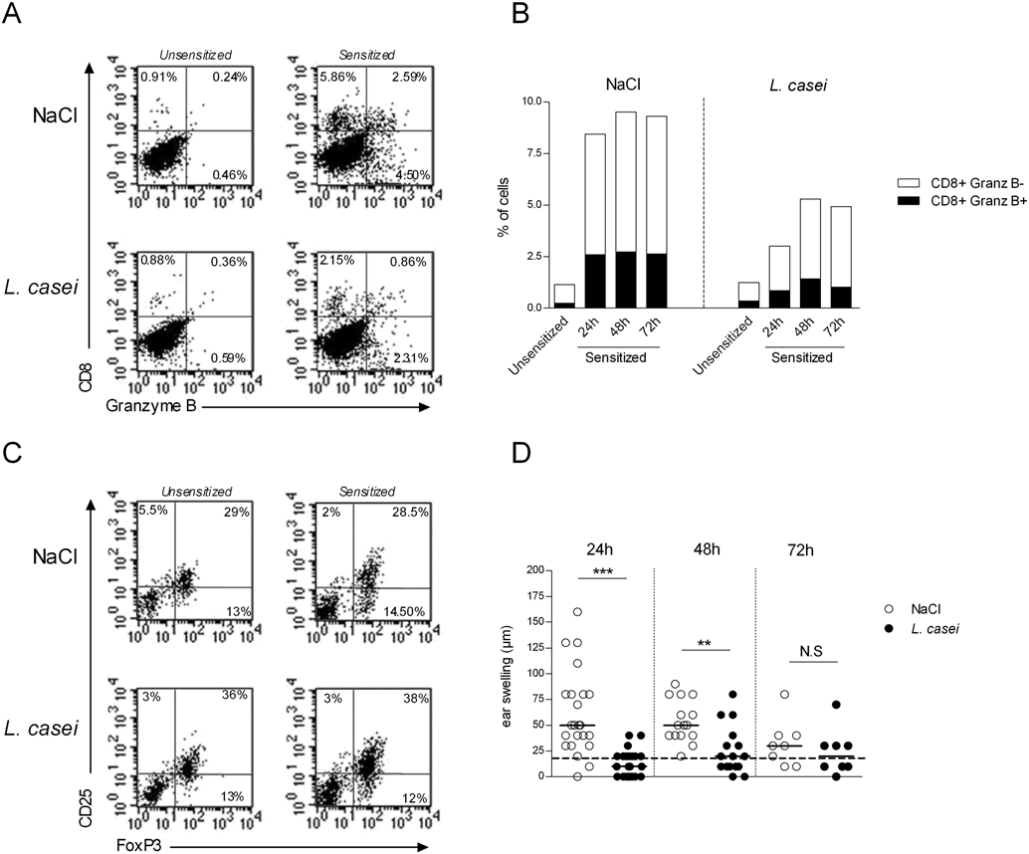
Effect of *L. casei* on CD8^+^ effectors and FoxP3^+^ regulatory T cells in the skin. Total cells were extracted from ears of either *L. casei* treated or NaCl mice that were either unsensitized, or DNFB-sensitized and harvested at 24 h, 48 h and 72 h post DNFB ear challenge. (A) Dot plot FACS analysis on gated CD45^+^ cells of ear cell suspensions staining for CD8 and granzyme B at 48 hr post challenge. (B) Histograms representation of the percentage of granzyme B^+^ CD8^+^ T cells (black portion) and granzyme B^−^ CD8^+^: white portion) among gated CD45^+^ leucocytes from NaCl- (left) and L. Casei (right) treated mice at various time point after ear challenge. (C) Dot plot FACS analysis of gated CD45^+^×CD4^+^ T cells after ear cell suspensions stained for CD4, CD25 and FoxP3 at 48 hr post DNFB challenge in naïve (left) or sensitized (right) mice, treated with NaCl (top) or *L. Casei* (bottom). (D) Corresponding ear swelling at 24 h, 48 h and 72 h post- DNFB challenge in DNFB sensitized mice, treated with NaCl (white circles) or *L. casei* (black circles).

The efferent symptomatic phase of the CHS response induced upon challenge is initiated by the recruitment into the challenged skin site of primed CD8^+^ T cells, which initiate skin inflammation by inducing apoptosis of kératinocytes [17], [18]. We thus examined the outcome of *L. casei* treatment on the number and kinetics of accumulation of cytolytic granzyme B^+^ CD8^+^ effectors into the skin, at various time after hapten challenge. Irrespectively of *L. casei* treatment, CD8^+^ T cells were hardly detectable in the skin of NaCl-treated control unsensitized mice at 48 h post ear challenge (**Fig 3A and B, ***left panels*). Ear challenge of sensitized mice with the hapten resulted in the rapid recruitment of CD8^+^ T cells including both granzyme B^+^ and granzyme B^−^ CD8^+^ CTL, which appeared within 24 hr after challenge and persisted for up to 72 hr (**Fig 3A** *upper right panel*, **and** **Fig 3B** *left*). Interestingly, both granzyme B^+^ and granzyme B^−^ subsets of CD8^+^ T cells were decreased in *L. casei*-treated mice (**Fig 3A** *lower right panel*, **Fig 3B** *right*).

### *L. casei* impairs the efficacy of naive CD8^+^ T cells to induce CHS

**Figure 4:**
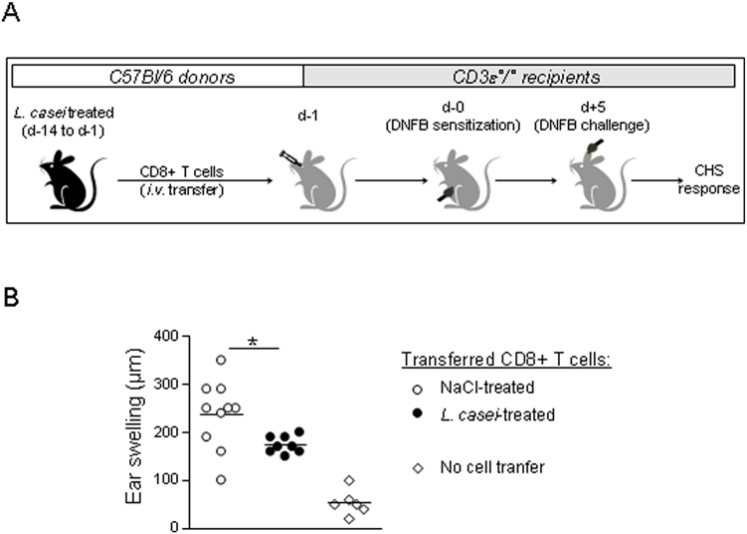
CD8^+^ T cells from *L. casei*-treated mice generate a weak CHS in CD3ε°/° recipients. (A) Naïve C57Bl/6 were treated daily for 2 weeks with either *L. casei* or NaCl 0.9%. On Day -1, pooled CD8^+^ T cells (10.10^6^) from pLN, mLN and spleens from each group of mice were transferred *i.v.* to naive CD3ε°/° recipient mice. On day 0 all CD3ε°/° recipients were DNFB sensitized and ear challenged with DNFB on Day 5. (B) Ear swelling at 48 h after DNFB-challenge was determined in CD3ε°/° recipients transferred with CD8^+^ T cells from either *L. casei*-treated (black circle) or NaCl-treated (white circle) C57Bl/6 donors as well as in untransferred control CD3ε°/° mice (white losange).

We recently reported that orally-induced tolerance of CD8^+^ T cell-mediated CHS to DNFB is initiated by a mucosal step of *in vivo* CD8^+^ T cell hyporesponsiveness, which impairs further differentiation of CD8^+^ T cells into CHS effectors [25]. To determine whether oral administration of *L. casei* could induce a similar conditioning of naive CD8^+^ T cells in lymphoid organs, we set up a model of CHS induced by adoptive transfer of CD8^+^ T cells into T cell deficient CD3ε°/° mice. MACS-sorted CD8^+^ T cells from naive C57Bl/6 donor mice that were treated daily for 14 days by oral administration of *L. casei* or NaCl, were transferred *i.v.* into naive syngenic CD3ε°/° recipients and the CHS response to DNFB was examined in the recipients (**Fig 4A**). As expected, un-reconstituted CD3ε°/° recipient mice were unable to mount a CHS response while reconstitution with CD8^+^ T cells from control NaCl treated donors induced a potent CHS response (mean ear swelling 237 µm±71). In contrast, skin inflammation in recipients of CD8^+^ T cells from *L. casei* treated donors, was significantly reduced (mean ear swelling 173.8 µm±18, **Fig 4B**). Thus, naive CD8^+^ T cells from systemic lymphoid organs conditioned by oral *L casei in vivo*, exhibited impaired ability to induce a CHS response *in vivo*.

### *L.* casei inhibition of CHS requires MHC class II-restricted CD4^+^ T cells but not CD1d-restricted NK-T cells

**Figure 5:**
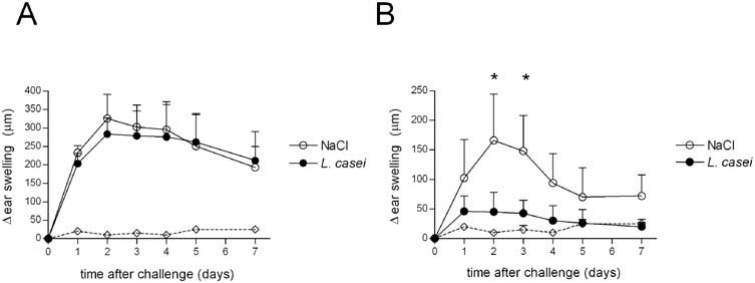
Inhibition of CHS by *L. casei* requires CD4^+^ T cells. Aβ°/° mice (A) and CD1d°/° mice (B) were treated daily from Day-14 before DNFB skin sensitization (Day 0) until the end of experiment with either live *L. casei* (black circle) or NaCl (white circle). All mice were sensitized on day 0 with DNFB and ear challenged on day 5 with DNFB. The CHS response was determined by ear swelling (µm) as indicated in Fig. 1 legend. Statistics (*p* values) were performed using the Mann Whitney two-sample and Student's *t*-test. Dotted lines represent ear swelling values after ear challenge of unsensitized mice.

CD4^+^ T cells are dispensable for the priming of CD8^+^ T cells mediating DNFB-specific CHS response, but contain regulatory T cells including MHC class II-restricted CD4^+^CD25^+^ Treg, which control skin inflammation [15], [16], [19], [21] and CD1d-restricted NK-T cells, which contribute to oral tolerance [26]. To determine whether *L. casei* inhibition of the CHS required regulatory T cells, we tested the outcome of oral *L. casei* treatment on CHS to DNFB in Aß°/° mice (deficient in MHC class-II restricted CD4^+^ T cells) and in CD1d°/° (deficient in NK-T cells). While *L. casei* treatment was able to reduce the intensity of the CHS response in CD1d°/° mice (**Fig 5A**) similarly than in wild type C57Bl/6 mice (**Fig 1A**), it was unable to control the hapten-specific skin inflammation in Aß°/° mice, (**Fig 5B**), indicating that regulation of DTH responses by *L. casei* depended on class II-restricted CD4^+^ Tregs but not on NK-T cells.

### *L. casei* treatment does not affect the *in vivo* suppressive function of nTregs in lymphoid organs of naive mice

**Figure 6:**
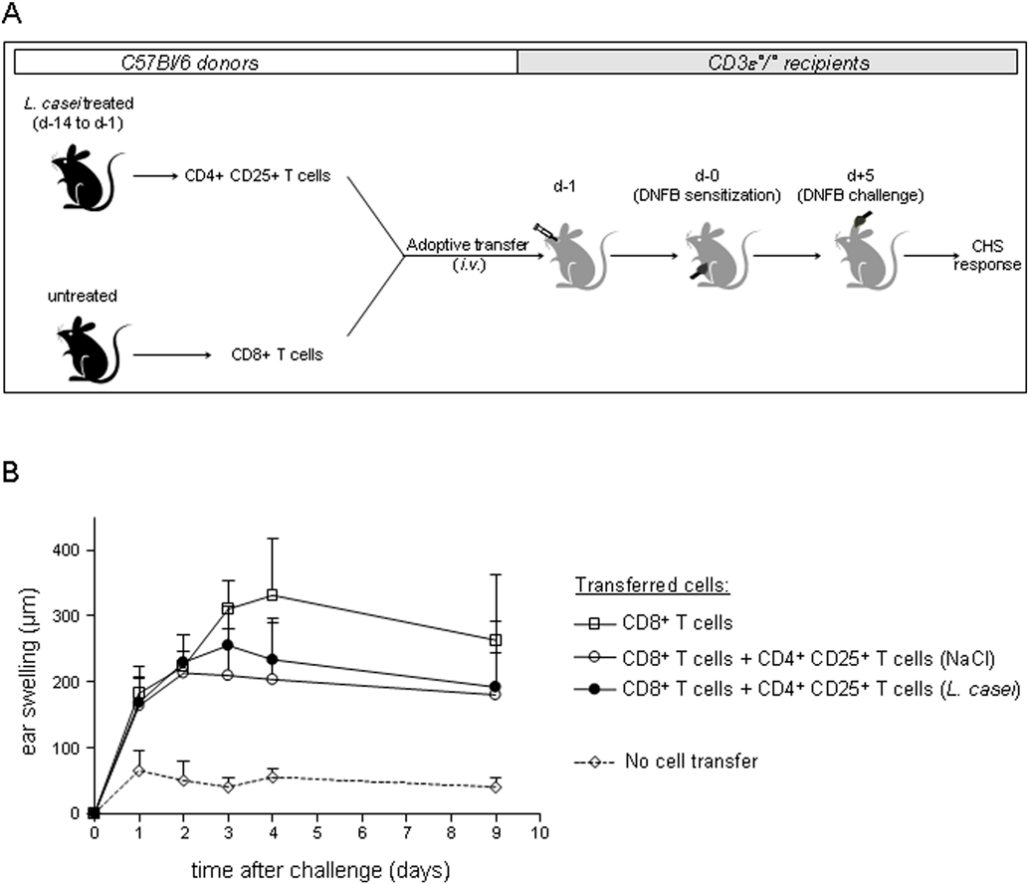
*In vivo* suppressive function of CD4^+^CD25^+^ Tregs from *L. casei*-treated mice. (A) Naïve C57Bl/6 were treated daily from d-14 to d-1 with either *L. casei* or NaCl. On d-1, CD4^+^ CD25^+^ T cells were purified from pooled pLN, mLN and spleens of each group of mice and transferred *i.v* together with naive purified CD8^+^ T cells into naive CD3ε°/° recipients. On day 0, untransferred and transferred CD3ε°/° recipients were DNFB-sensitized and challenged 5 days later with DNFB, as described in Fig 1 legend. (B) Ear swelling was determined at various time after challenge in CD3ε°/° that were either untransferred (dotted lines) or transferred with CD8^+^ T cells alone (white squares), or with CD8^+^ T cells together with CD4^+^CD25^+^ T cells from *L. casei*- treated (black circle) or NaCl-treated (empty circles) donors.

To determine whether *L. casei* treatment affected the pool of natural CD4^+^CD25^+^ Tregs of lymphoid organs at the steady state (*i.e.* unsentitized mice), we set up an adoptive co-transfer model of CD4^+^CD25^+^ Tregs and CD8^+^ T cell into T cell deficient CD3ε°/° mice. MACS-sorted CD4^+^CD25^+^ T cells harvested from naive C57Bl/6 donor mice treated by daily oral administration of *L. casei* or NaCl as control for 14 days, were co-transferred with CD8^+^ T cells from naive C57Bl/6 into syngenic CD3ε°/° recipients one day before skin sensitization and the CHS response to DNFB was examined after ear challenge with DNFB (**Fig 6A**). As described above (**Fig 4B**), CD3ε°/° recipient mice mounted a CHS response to DNFB only upon CD8 reconstitution, with a mean ear swelling of 330 µm±85 at day 4 after challenge, that was sustained for up to day 9 (**Fig 6B**). Co-transfer of Tregs cells from either NaCl- or *L. casei*- treated donors resulted in a similar 30% (p = 0.0076) reduction of skin inflammation with mean ear swelling values at 96 hr post challenge of 220 µm±92 and 230 µm±57, respectively. These data demonstrate that daily oral exposure to *L. casei* in a naive host is not capable to affect the potential of natural CD4^+^ CD25^+^ Treg of lymphoid organs to suppress a CHS response.

### *L casei* affects the frequency of CD4^+^FoxP3^+^Treg in the skin

Analysis of the frequency of Tregs in the skin cells was carried out in naive mice as well as after DNFB challenge of sensitized C57Bl/6 mice. At 48 hr post hapten skin challenge, control unsensitized mice exhibited a high proportion of FoxP3^+^CD4^+^ T cells (*i.e.* 42% of total CD4^+^ T cells), among which roughly 2/3 expressed the CD25 molecule (**Fig 3C**, *upper left*); sensitization did not affect the frequency of FoxP3^+^CD4^+^ Treg present in the skin at 48 hr after challenge (**Fig 3C**, *upper right*). Alternatively, *L. casei* treatment increased the frequency of skin resident CD4^+^CD25^+^FoxP3^+^ Tregs in control unsensitized mice, to a level that was maintained up to 48 hr post challenge in sensitized mice (**Fig 3C**, *right panels*). These effects correlated with a dramatic inhibition of the CHS response in *L. casei*-treated mice, as confirmed in the same experiment (**Fig 3D**).

### *L. casei* treatment increases IL-10 production by Tregs in sensitized mice

**Figure 7:**
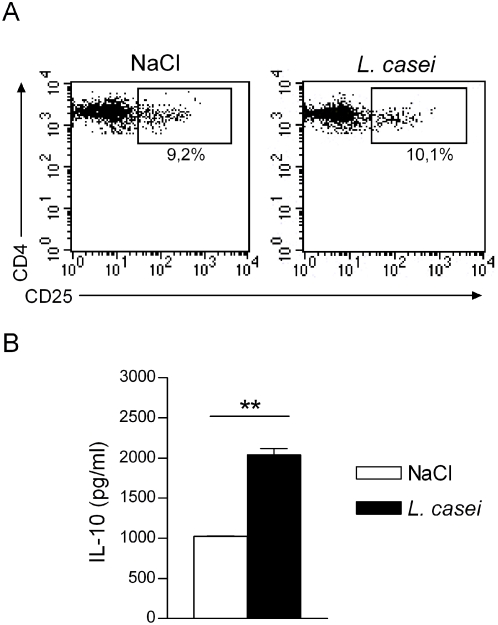
Increased IL-10 production by CD4^+^CD25^+^ T cells from *L. casei* treated mice. C57Bl/6 mice were treated with *L. casei* or NaCl from Day-14 before DNFB skin sensitization until Day+5 post-sensitization. (A) on Day 5, spleen cell suspensions from *L. casei*-treated (right panel) or NaCl–treated (*left panel*) mice were stained for FACS analysis. The dot plot shows the percentages of CD4^+^CD25^+^ cells on gated CD4^+^ T cells. (B) On Day 5, CD4^+^CD25^+^ T cells purified from spleen of *L. casei* treated (black bar) or NaCl- treated (white bar) mice were restimulated *in vitro* with soluble anti-mouse CD3+mIL-2 and 72 hr culture supernatants were titrated for IL-10 by Elisa.

We next asked whether *L. casei* treatment could have effect on antigen experienced- CD4^+^CD25^+^ Tregs. To test this hypothesis, CD4^+^CD25^+^ T cells were isolated from *L. casei*- or NaCl-treated mice on day 5 after DNFB sensitization and tested *ex-vivo* for IL-10 production in response to *in vitro* stimulation with anti-CD3 mAb with IL-2. CD4^+^CD25^+^ T cells from NaCl-treated DNFB-sensitized mice produced IL-10 upon polyclonal *in vitro* stimulation with anti-CD3 and treatment with *L. casei* did not affect percentage of CD4^+^CD25^+^ cells of either naïve (*not shown*) or day 5-sensitized (**Fig 7A**) C57Bl/6 mice. However, *L. casei* treatment resulted in a 3 fold increase in IL-10 production by CD4^+^CD25^+^ T cells from sensitized mice, in response to *ex vivo* stimulation with anti-CD3+IL-2 (**Fig 7B**). These data indicate that *L. casei* treatment is capable to promote the activation of Ag-experienced CD4^+^CD25^+^ Tregs and increases their ability to produce IL-10.

## Discussion

Our data demonstrate that oral administration of *L. casei* DN-114 001 is able to alleviate skin DTH responses mediated by Ag-specific CD4^+^ or CD8^+^ T cells. This is reminiscent of recent studies showing that lactic acid bacteria decrease the Th1-type CD4 responses to type II collagen in a mouse model of arthritis [27]. In the model of CHS to the hapten DNFB, we show here that preventive oral treatment with *L. casei* alleviates skin inflammation. *L. casei* treatment affected only on the symptomatic phase of the disease, induced shortly after challenge by the recruitment of pathogenic CD8 CTL at the site of hapten challenge [15], [17]. Indeed, *L. casei* did not impair the *in vivo* priming and differentiation of hapten-specific cytolytic CD8^+^ CHS effectors during the afferent phase induced by skin sensitization with DNFB. These data thus emphasized that anti-inflammatory effect of *L. casei* on skin inflammation does not merely result from an immunosuppressive effect on T cells. Alternatively, *L. casei* decreased the recruitment of pathogenic CD8^+^ effectors into the skin at the site of challenge. We previously reported that the cytolytic function of CD8^+^ T cells via the perforin and Fas/Fas-L pathways is crucial to the initiation of skin lesions of ACD [17]. The fact that both Granzyme B-expressing and Granzyme B negative CD8^+^ T cells recruitment into the challenged skin was inhibited in *L. casei*-treated mice indicated that the probiotic affected the mobilization of both CTL effectors and non cytolytic activated CD8^+^ T cells. Along these lines, CD8^+^ T cells from *L. casei*- treated but unsensitized mice have a reduced capacity to generate a CHS response, as demonstrated in the CD8 transfer model into T cell-deficient recipient although the same frequency of hapten-specific IFNγ producing cells were generated in the recipients (*not shown*). This underscores the intriguing possibility that oral exposure to *L. casei* has an impact on the pool of naive hapten-reactive CD8^+^ T cells in lymphoid organs that conditions their subsequent ability to differentiate into CHS effectors. This observation is reminiscent to our recent finding that orally induced tolerance of CHS to DNFB, is initiated by a mucosal step of hapten presentation by plasmacytoid DC in liver and MLN, which induces partial deletion of hapten-specific CD8 T cells [25]. It is possible that CD8^+^ T cell conditioning by oral exposure to *L. casei* is mediated via pDC, inasmuch as these cells are able to negatively control the CHS response (*personnal data*). Alternatively, *L. casei* may affect the homing potential of CD8^+^ T cells into inflamed tissues. Indeed, several chemokines are implicated in CD8^+^ T cell recruitment during the CHS response [28]. In this respect, up-regulation of CCL5/RANTES on CD8^+^ T cells from DNFB sensitized mice was shown to contribute to the recruitment of additional bystander CD8^+^ T cells [28]. It may thus be hypothesized that *L. casei* controls the ability of CD8 to produce or respond to CCL5/RANTES.

The observation that *L. casei*'s inhibitory effect on the CHS response depends on the presence of MHC class-II restricted CD4^+^ T cells indicates that *L. casei* may also affect the capacity of Treg to control the CHS response. We and others have demonstrated that Treg including CD4^+^CD25^+^ natural Tregs control the *in vivo* priming of the hapten-specific CD8^+^ effectors in lymphoid organs [15], [16]. In addition, Tregs are recruited into the inflamed skin as CD4^+^FoxP3^+^ T cells that acquire the CD25 molecule (Fig 3 and data *not shown*). In this study we found that splenic CD4^+^CD25^+^ T cells from *L. casei*-treated and DNFB sensitized mice exhibit a higher capacity to produce IL-10, indicating that the probiotic may activate Ag-induced Tregs in lymphoid organs. In addition, we observed an increased number of CD4^+^CD25^+^FoxP3^+^ Tregs in the skin of naive *L. casei*-treated mice. Since Treg have be shown to suppress CHS by blocking influx of effector CD8^+^ T cells into inflamed tissue [29], these data suggest that *L. casei* may promote the homing and/or the conversion of nTregs in normal skin. To ask whether *L. casei* could be able to promote *de novo* conversion of nTreg *in vivo*, we performed an adoptive transfer experiment by injecting Foxp3-eGFP negative CD4^+^ T cells from Foxp3-*egfp* transgenic mice into RAG2°/° recipient mice, that were subsequently treated or not with *L. casei* for 3 months. Although, neoconversion of Foxp3^+^ nTreg was observed in the mesenteric lymph nodes and intestinal lamina propria, and to a much lesser degree in systemic lymphoid organs, similar neoconversion occurred irrespective of oral exposure of recipients to *L. casei (not shown)*. Thus it appears unlikely that at the steady state, the probiotic could have induced de novo generation of nTreg in the skin. So far we don't know how oral gavage with a probiotic can affect Treg in skin at the homeostatic state, i.e. in the absence of inflammatory signal. It is possible that components of the probiotics, (such as nucleic acid or peptidoglycans) may enter the intestinal lamina propria, circulate via blood and reach peripheral non mucosal tissues such as the skin. There, they could act directly or indirectly by enhancing the survival and/or proliferation of resident Treg possibly via binding to Toll-like receptors[30] or other commensal sensoring receptors. Another possibility would be that probiotic components circulating via the blood may reach lymphoid organs and condition DC/T cell interactions leading to enhanced expression of skin homing receptors on T cells.

How oral administration of probiotic bacteria can affect T cell mediated inflammation in remote skin tissue remains unclear. Studies have shown that lactic acid bacteria can reduce intestinal inflammation in IL-10°/° and TNBS models of colitis even when administered by the parenteral and the subcutaneous routes, respectively [7], [31]. It is possible that components of the bacteria circulating via the blood flow or via lymph may reach gut-draining and non draining lymphoid organs [32], exert their effect on Ag-presenting DC [33], [34]. In this respect, recent studies showed that DC pre-incubated with lactic acid bacteria can alleviate colitis as efficiently as after systemic delivery of the probiotic [7]. Moreover, *L. casei* was shown to promote the ability of DC to expand T cells with suppressive function[35]. Whether *L. casei* may use DC to behave as biological adjuvants for distinct subsets of Treg, including Foxp3^+^ nTreg or Ag-induced Treg and whether this probiotic may act by promoting their expansion, survival and/or homing properties remains an important issue to be further elucidated.

Taken together our results show that *L. casei* DN-114001 administered orally is able to alleviate allergic skin inflammation, without causing immuno-suppression, but via mechanisms that include control the pathogenic function of CD8^+^ T cells and require Tregs.
